# Dietary animal and plant protein intakes and their associations with obesity and cardio-metabolic indicators in European adolescents: the HELENA cross-sectional study

**DOI:** 10.1186/1475-2891-14-10

**Published:** 2015-01-21

**Authors:** Yi Lin, Theodora Mouratidou, Carine Vereecken, Mathilde Kersting, Selin Bolca, Augusto César F de Moraes, Magdalena Cuenca-García, Luis A Moreno, Marcela González-Gross, Jara Valtueña, Idoia Labayen, Evangelia Grammatikaki, Lena Hallstrom, Catherine Leclercq, Marika Ferrari, Frederic Gottrand, Laurent Beghin, Yannis Manios, Charlene Ottevaere, Herman Van Oyen, Denes Molnar, Anthony Kafatos, Kurt Widhalm, Sonia Gómez-Martinez, Ligia Esperanza Díaz Prieto, Stefaan De Henauw, Inge Huybrechts, On behalf of the HELENA study group

**Affiliations:** Department of Public Health, Faculty of Medicine and Health Sciences, Ghent University, UZ – 4K3, De Pintelaan 185, B-9000 Ghent, Belgium; Growth, Exercise, Nutrition and Development (GENUD) Research Group, Faculty of Health Sciences, University of Zaragoza, c/ Perdro Cerbuna 12, 50009 Zaragoza, Spain; Forschungsinstitut für Kinderernährung, Research Institute of Child Nutrition, Dortmund, Rheinische Friedrich-Wilhelms, Universität Bonn, Heinstück 11, 44225 Dortmund, Germany; Laboratory for Bioinformatics and Computational Genomics (Biobix), Faculty of Bioscience Engineering, Ghent University, Coupure Links 653, B-9000 Ghent, Belgium; Department of Medical Physiology, School of Medicine, Granada University, Avenida Madrid 12, 18012 Granada, Spain; ImFine Research Group, Department of Health and Human Performance, Faculty of Physical Activity and Sport Sciences (INEF), Universidad Politécnica de Madrid, Martin Fierro 7, 28040 Madrid, Spain; Department of Nutrition and Food Science, University of the Basque Country, Paseo de la Universidad 7, 01006 Vitoria-Gasteiz, Spain; Division of Public Health Sciences and Division of Sociology, School of Health, Care and Social Welfare, Märlardalens University, Box 883, 72123 Västerås, Sweden; Agricultural Research Council—Food and Nutrition Research Centre, Via Ardeatina 546, 00178 Rome, Italy; Inserm U995, Faculté de Médecine, Université de Lille, Lille, France; Department of Nutrition and Dietetics, Harokopio University, 70, El Venizelou Ave, 17671 Kallithea, Athens Greece; Scientific Institute of Public Health, J. Wytsmanstraat 14, 1050 Brussels, Belgium; Department of Pediatrics, University of Pécs, Pécs, József A. u. 7, 7623 Pécs, Hungary; Preventive Medicine and Nutrition Unit, University of Crete, School of Medicine, Heraklion, Crete Greece; Department of Pediatrics, Private Medical University, Strubergasse 21, 5020 Salzburg, Austria; Immunonutrition Research Group, Department of Metabolism and Nutrition, Institute of Food Science, Technology and Nutrition (ICTAN), Spanish National Research Council (CSIC), 10 Antonio Novais street, 28040 Madrid, Spain; Dietary Exposure Assessment group, International Agency for Research on Cancer, 150 Cours Albert Thomas, 69372 Lyon, CEDEX 08 France; Research Foundation – Flanders (FWO), Egmontstraat 5, 1000 Brussels, Belgium; YCARE (Youth/Child and cArdiovascular Risk and Environmental) Research Group, Department of Preventive Medicine School of Medicine of the University of Sao Paulo, 01246-903 São Paulo, Brazil; Department of Nutrition and Dietetics, Faculty of Health Care Vesalius, University College Ghent, Keramiekstraat 80, B-9000 Ghent, Belgium; Centre d’Investigation Clinique, CIC-PT-1403-Inserm-CH&U, 59037 Lille, France; Department of Preventive Medicine, School of Medicine of the University of São Paulo, São Paulo, Brazil

**Keywords:** Protein intake, Adolescence, Body composition, Biomarkers, HELENA study

## Abstract

**Background:**

Previous studies suggest that dietary protein might play a beneficial role in combating obesity and its related chronic diseases. Total, animal and plant protein intakes and their associations with anthropometry and serum biomarkers in European adolescents using one standardised methodology across European countries are not well documented.

**Objectives:**

To evaluate total, animal and plant protein intakes in European adolescents stratified by gender and age, and to investigate their associations with cardio-metabolic indicators (anthropometry and biomarkers).

**Methods:**

The current analysis included 1804 randomly selected adolescents participating in the HELENA study (conducted in 2006–2007) aged 12.5-17.5 y (47% males) who completed two non-consecutive computerised 24-h dietary recalls. Associations between animal and plant protein intakes, and anthropometry and serum biomarkers were examined with General linear Model multivariate analysis.

**Results:**

Average total protein intake exceeded the recommendations of World Health Organization and European Food Safety Authority. Mean total protein intake was 96 g/d (59% derived from animal protein). Total, animal and plant protein intakes (g/d) were significantly lower in females than in males and total and plant protein intakes were lower in younger participants (12.5-14.9 y). Protein intake was significantly lower in underweight subjects and higher in obese ones; the direction of the relationship was reversed after adjustments for body weight (g/(kg.d)). The inverse association of plant protein intakes was stronger with BMI z-score and body fat percentage (BF%) compared to animal protein intakes. Additionally, BMI and BF% were positively associated with energy percentage of animal protein.

**Conclusions:**

This sample of European adolescents appeared to have adequate total protein intake. Our findings suggest that plant protein intakes may play a role in preventing obesity among European adolescents. Further longitudinal studies are needed to investigate the potential beneficial effects observed in this study in the prevention of obesity and related chronic diseases.

## Introduction

The prevalence of overweight (OW) and obesity (OB) in adolescents, defined on the basis of body mass [1], has increased rapidly worldwide. In 2010, the estimated prevalence of OW and OB in European children and adolescents was approximately 38%, including 10% OB [2]. As a consequence of OB-related co-morbidities, over 20000 children suffer from type 2 diabetes and more than 400000 have impaired glucose levels [2]. Childhood OW and OB both influence long-term health and evidence suggest an association with coronary events and mortality later in life [3, 4].

Nutrition during the early years of life is a critical factor of OB in adolescence further impacting on adulthood OW and OB, and the consequences of chronic diseases [5, 6]. High protein intakes were reported to improve cardiovascular risk factors including abdominal OB, dyslipidemia, glucose intolerance, and hypertension in European children (5–18 y) [7]. Previous randomised trials [8, 9] suggest that a high-protein diet defined as ≥20% of total energy lowers the risk of OW and promotes weight maintenance among adolescents [10]. The association between dietary protein intake and adolescent OW and OB has mainly been investigated in relation to its increased thermic effect and satiety when compared to fats and carbohydrates [9, 11]. Others, however, have reported that higher protein content in the diet did not confer any benefit in the treatment of OB among children 9–18 y old [12].

The debate on protein sources is still ongoing, addressing the nutritional quality of dietary proteins based on their amino acids composition. The protein quality or biological value of proteins from animal sources is high, whereas most plant proteins lack one or more essential amino acids and are therefore considered as incomplete proteins. What some seem to be concerned with is that the majority of high-protein foods are significant sources of fat and/or sugar as well (such as meat and meat products, cheese, and dairy desserts), and should therefore be carefully selected. Hermanussen *et al.* reported a positive correlation between the energy contribution of animal proteins to the diet and the body mass index (BMI) in adolescents [13]. On the other hand, Bradlee *et al.* found no association between OB and meat consumption among adolescents [14], while, plant-based diets were inversely associated with normal BMI in children in Hermanussen’s study [13]. A Western dietary pattern high in animal sources is associated with an increased risk of metabolic syndrome (MetS) [15, 16], whereas diets high in fruits, vegetables and whole grains are associated with a decreased risk [17]. Evidence showed that plant protein, soy in particular, can bind phytoestrogen compounds to stimulate lipid metabolism resulting in a better blood profile, by lowering total cholesterol (TC), triglyceride (TG), low-density lipoprotein-cholesterol (LDL-C) and reducing insulin resistance [18, 19].

The aim of the current study was to evaluate total, animal and plant protein intakes in European adolescents and to investigate their association with cardio-metabolic indicators (anthropometry: BMI z-score and body fat percentage (BF%); and biomarkers: TC, TG, LDL-C, very LDL-C (VLDL-C), high-density lipoprotein cholesterol (HDL-C), C-reactive protein (CRP), glucose, insulin and leptin).

## Methods

### Survey population

The Healthy Lifestyle in Europe by Nutrition in Adolescence-Cross Sectional Study (HELENA-CSS) is a European Commission funded project on lifestyle and nutrition among adolescents from 10 cities of European countries: Stockholm, Athens, Heraklion, Rome, Zaragoza, Ghent, Lille, Dortmund,Vienna, and Pecs that ran between October 2006 and December 2007. Due to logistical reasons, adolescents from Heraklion and Pecs were excluded for the dietary intake assessments. A multi-stage random cluster sampling procedure was used to select 3528 adolescents, stratified by geographical location, age and socioeconomic status (SES). Schools were randomly selected after stratification to guarantee diversity of the sample in culture and SES.

Male and female adolescents, aged 12.5-17.5 y, not participating simultaneously in a clinical trial, free of any acute infection lasting less than 1 week before inclusion year, and who provided two 24-h recall interviews with valid information and complete anthropometric measurements, were included in the final analysis of the current study. Details on sampling procedures, study design and non-respondents have been reported elsewhere [20, 21].

The study was approved by the Research Ethics Committees of each city involved. Written informed consent was obtained from the adolescents’ parents and the adolescents themselves [22].

### Dietary intake assessment

Two non-consecutive computerised 24-h dietary recalls (HELENA-DIAT), instructed by dieticians/researchers, were used to collect food consumption data. During interviews, adolescents were allowed to ask questions and following completion the recall was checked for completeness. Each participant was asked to complete the recall twice in a time-span of 2 weeks during the school time.

HELENA-DIAT is a self-administered computer program based on the Young Adolescents’ Nutrition Assessment on Computer (YANA-C) [23], consisting of a single computerised 24-h recall with a structured program based on six meal occasions. The validated YANA-C [23], was designed to obtain a detailed description and quantification of foods consumed, and eventually included about 800 food items hierarchically organized in 25 food groups, and about 300 colored photograph sets of foods in different portions [24, 25].

Dietary intakes were linked to the German Food Code and Nutrient DataBase (BLS (Bundeslebensmittelschlüssel), version II.3.1, 2011) [26]. However, the estimated percentage of animal and plant protein intakes were calculated by linking the 24-h recall food consumption data to the Belgian NUBEL [27], the Dutch NEVO [28] and the USDA [29] food composition databases which used the Kjeldahl method for analysing protein [30], because no differentiation was made between plant and animal proteins in the BLS database. Protein intakes were calculated in absolute terms (g/d) and relative terms (energy percentages (E%); per kg body weight).

Under-reporters, excluded in the current study, were considered as individuals with a ratio of energy intake over estimated basal metabolic rate lower than 0.96 [31].

### Anthropometric measurements

Weight (kg) and height (m) were measured in underwear and barefoot to the nearest 0.1 kg and 0.1 cm, respectively, by trained researchers. BMI was calculated as weight (kg)/height (m^2^). Participants were classified into four BMI categories according to the International Obesity Task Force (IOTF) cut-offs for adolescents [1]: equivalent to underweight (UW) (<18.5 kg/m^2^), normal weight (NW) (18.5-24.9 kg/m^2^), OW (25.0-29.9 kg/m^2^), and OB (≥30.0 kg/m^2^). Standard deviation score of BMI (BMI z-score) was calculated using the lmsGrowth method [32]. The cut-off of BMI z-score [33]: UW (<-2), NW (-2 -1), OW (>1) and OB (>2). Skinfold thickness was measured to the nearest 0.2 mm in triplicate [34]. The same trained investigators made all measurements (inter-rater reliability >95 %). BF% was calculated using Slaughter’s equations [35]. More details about the anthropometric measurements are given in a previous manuscript [34]. Physical maturations were examined by a physician during a medical examination to determine the pubertal status based on Tanner stages [36]. The final physical maturations were classified into three categories: pre-pubertal (stage 1); pubertal (stage 2 to 4) and post-pubertal (stage 5).

### Blood samples

Blood samples were collected in a randomly selected subsample of the total HELENA-CSS. Adolescents who agreed to be involved in the blood sampling were asked to fast after 8 pm on the previous day. Fasting blood samples, information of adolescents’ medical history and recent acute diseases were collected by venipuncture between 8–10 a.m. at schools or hospitals by a medical doctor, A blood sampling questionnaire was completed by the participants for the purposes of assessing fasting status, acute infection, allergies, smoking, vitamin and mineral supplements, and medication. A specific handling, transport and traceability system for biological samples was developed for the HELENA study. All samples were analyzed centrally. The blood sampling procedure has been described elsewhere [37].

### Physical activity

Physical activity (PA) was assessed for 7 days by an uniaxial accelerometer (Actigraph GT1M), described previously [38]. At least 3 days of recording with a minimum of 8 hours’ registration per day was set as an inclusion criterion. PA, used in the current study, was categorized in the following categories: at least 1 hour of PA per day, no PA or less than 1 hour of PA per day.

### Statistical analysis

Descriptive data is presented as means with standard deviation or frequency distributions. Energy and total, animal and plant protein intakes were corrected for within-person variation using the Multiple Source Method (MSM), which is suitable for estimating population’s usual intakes [39]. Statistical differences for total energy and total, animal and plant protein intakes between subgroups (gender and age) were assessed using the Student *T*-test and ANOVA.

GLM multivariate analysis was used to investigate the associations of indicators (dependent variables) with animal and plant protein intakes, and animal (E%) and plant (E%) energy percentages (independent variables) through three models (stepwise approach): (1) model 1 = unadjusted model; (2) model 2 = model 1 + adjusted for fat intake; (3) model 3 = model 2 + further adjusted for PA, confounding factors and interactions, and controlling for the country clustering effect. Potential confounding factors including age (younger group (12.5-14.9 y) and older group (15.0-17.5 y)), gender, tanner stage (pre-puberty, puberty and post-puberty) and two-way interactions between potential confounding factors and independent variables were included in the model 3. Anthropometry and serum biomarkers were investigated separately. In addition, animal and plant protein intakes, and the energy percentage (E%) from animal and plant protein were examined in a separate model due to colinearity.

All statistical analysis were performed using the statistical software SPSS for Windows version 18 (SPSS Inc, Chicago, IL, USA). Results were considered statistically significant at α two-tailed level of 0.05.

## Results

A total of 1804 out of 3528 adolescents (47% males) from 8 centres with valid and complete dietary data and measurements of weight and height were included in the analysis (Table 1). 74% participants were classified in tanner stage 2–4, including 7% in tanner 2, 24% in tanner 3 and 41% in tanner 4. In total 279 adolescents were classified as OW and OB. Mean BMI z-score for both genders was in the NW range. Females had higher BF %, but lower BMI z-score compared to males. Furthermore, higher serum lipid profiles and leptin levels were found in females.

**Table 1:** **Anthropometric characteristics and levels of obesity-related biomarkers in adolescents participating in the HELENA-CSS**

	Total	Males	Females
Total participants (n)	1804	855	949
Age (y) (mean (range))	14.7 (12.5-17.4)	14.8 (12.5-17.4)	14.7 (12.5-17.4)
12.5-14.9 y (n)	1032	481	551
15.0-17.5 y (n)	772	374	398
Tanner Stage (n = 1752)	n (%)		
Tanner 1	9 (0.514)	9 (1.1)	0 (0.0)
Tanner 2-4	1294 (73.9)	614 (74.2)	680 (73.5)
Tanner 5	449 (25.6)	204 (24.7)	245 (26.5)
Weight status (n = 1804)^μ^			
Underweight	142 (7.9)	58 (6.8)	84 (8.9)
Normal weight	1383 (76.7)	649 (75.9)	734 (77.3)
Overweight	222 (12.3)	114 (13.3)	108 (11.4)
Obesity	57 (3.2)	34 (4.0)	23 (2.4)
	Mean (SD)		
Anthropometry			
BMI z-score (n = 1804)	0.270 (1.1)	0.358 (1.1)	0.190 (1.0)
BF% (n = 1764)	22.0 (8.6)	18.4 (9.1)	25.1 (6.8)
Biomarkers			
TC (mg/dL) (n = 552)	159.1 (27)	151.9 (24.9)	165.8 (27.1)
TG (mg/dL) (n = 552)	67.6 (31.1)	64.5 (31.5)	70.5 (30.5)
LDL-C (mg/dL) (n = 552)	92.6 (24.2)	89.0 (23.2)	96.0 (24.7)
VLDL-C (mg/dL) (n = 552)	13.5 (6.2)	12.9 (6.3)	14.1 (6.1)
HDL-C (mg/dL) (n = 552)	55.6 (10.3)	53.3 (9.3)	57.8 (10.7)
CRP (mg/L) (n = 524)	1.2 (4.0)	1.5 (5.5)	0.841 (1.3)
Glucose (mg/dL) (n = 552)	90.1 (7.0)	91.9 (7.2)	88.5 (6.4)
Insulin (μlU/mL) (n = 545)	9.5 (6.0)	9.0 (6.6)	10.0 (6.6)
Leptin (ng/mL) (n = 518)	18.5 (21.9)	8.1 (12.9)	27.5 (24.1)

SD, standard deviation; BMI, body mass index; BF%, body fat percentage; TC, total cholesterol; TG, triglycerides; LDL, low-density lipoprotein- cholesterol; VLDL-C, very low-density lipoprotein- cholesterol; HDL-C, high-density lipoprotein- cholesterol; CRP, c-reactive protein.

^μ^BMI categories is classified based on the International Obesity Task Force cut-offs, underweight: <18.5 kg/m^2^, normal weight: 18.5-24.9 kg/m^2^, overweight: 25.0-29.9 kg/m^2^, obesity: ≥30.0 kg/m^2^.

### Total energy and total, animal and plant protein intakes

Median total protein contributing to energy intake was 15.5%. Average total protein intakes exceeded the World Health Organization (WHO) recommendations (10.0 – 15.0% of the total energy intake) [40] and the estimated average requirements (EAR) and population reference intake (PRI) of the European Food Safety Authority (EFSA) (EAR: 0.66 g/(kg.d) for both genders; PRI: males, 0.70-0.74 (g/(kg.d), and females, 0.67-0.72 g/(kg.d)) [41] (Table 2). All but one adolescent met the EAR, while, fourteen and two adolescents did not reach the WHO recommendations for protein intakes and the PRI, respectively.

**Table 2:** **Percentile of total protein intakes and the number of the subjects below the recommendations of European food safety authority in the European adolescents**

Characteristics	N	Total protein (g/d)			Total protein (g/(kg.d))			The number of subjects below the recommendations	
		25%	50%	75%	25%	50%	75%	EAR ^μ^	PRI ^μ^
Total	1804	76	91	109	1.3	1.6	2.0	1	2
Gender									
Males	855	90	106	127	1.5	1.8	2.3	0	0
Females	949	68	80	94	1.2	1.5	1.8	1	2
Age									
12.5-14.9 y	1032	74	90	108	1.4	1.7	2.1	1	1
15.0-17.5 y	772	77	94	112	1.3	1.6	1.9	0	1

EAR: estimated average requirement; PRI: population reference intake.

^μ^EAR: 0.66 g/(kg.d) for both genders; PRI : males, 0.70-0.74 g/(kg.d) and females, 0.67-0.72 g/(kg.d).

Mean total protein intake (384 kcal/d) contributed 15.8% to total energy intake. Mean animal protein intakes were the main contributor (59%) to total protein intakes, as opposed to mean plant protein (Table 3). Total and plant protein intakes were significantly lower in females and the younger group. Body weight adjusted total protein intakes and E% from total protein were significantly lower in the older group. Total energy, total and animal protein intakes and total protein (E%) were higher in obese adolescents than non-obese ones. More specifically, body weight adjusted total protein intake (g/(kg.d)) was significantly lower in OB, and higher in UW peers.

**Table 3:** **Estimated means of energy, total, animal and plant protein intakes, and energy percentage of protein intakes of adolescents participating in the in HELENA-CSS stratified by gender, age, tanner and BMI category**

Characteristics	N	Energy (kcal/d)	Total protein (g/d)	Total protein (g/(kg.d))	Animal protein (g/d)	Plant protein (g/d)	Total protein	Plant protein
							% energy contributing to total energy intake	
		Mean intake (SD)						
Total	1804	2450 (637)	96 (28)	1.7 (0.6)	58 (23)	38 (13)	15.8 (2.8)	6.2 (1.3)
Gender								
Males	855	2792 (655)	110 (29)	1.9 (0.6)	66 (24)	43 (13)	15.9 (3.0)	6.2 (1.3)
Females	949	2141 (428)*	83 (20)*	1.6 (0.5)*	50 (18)*	33 (10)*	15.6 (2.7)	6.3 (1.3)
Age								
12.5-14.9 y	1032	2358 (637)	94 (28)	1.8 (0.6)	57 (22)	37 (12)	16.1 (2.9)	6.2 (1.4)
15.0-17.5 y	772	2752 (713)**	98 (29)**	1.6 (0.5)**	58 (23)	39 (12)**	15.4 (2.8)**	6.2 (1.2)
Weight status								
Underweight	142	2443 (631)	94 (28)	2.2 (0.7)	56 (21)	39 (12)	15.5 (2.7)	6.3 (1.2)
Normal weight	1383	2458 (635)	96 (28)^a^	1.8 (0.6)^a^	58 (22)	38 (13)	15.7 (2.8)	6.2 (1.3)
Overweight	222	2397 (636)	96 (29)^ab^	1.4 (0.4)^ab^	59 (24)	37 (11)	16.2 (3.0)^b^	6.2 (1.3)
Obesity	57	2476 (701)	102 (33)^abc^	1.2 (0.4)^ab^	63 (27)	38 (12)	16.5 (3.1)^b^	6.2 (1.2)

SD, standard deviation.

*Mean value was significantly different between males and females by Student *T*- test (*P* < 0.05).

**Mean value was significantly different from the young group (12.5-14.9 y) by Student *T*- test (*P* < 0.05).

^a^Mean value was significantly different from underweight by ANOVA, (*P* < 0.05, Bonferroni correction.

^b^Mean value was significantly different from normal weight by ANOVA, (*P* < 0.05, Bonferroni correction).

^c^Mean value was significantly different from overweight by ANOVA, (*P* < 0.05, Bonferroni correction).

### Associations between total, animal and plant protein intakes and cardio-metabolic indicators

**Figure 1:**
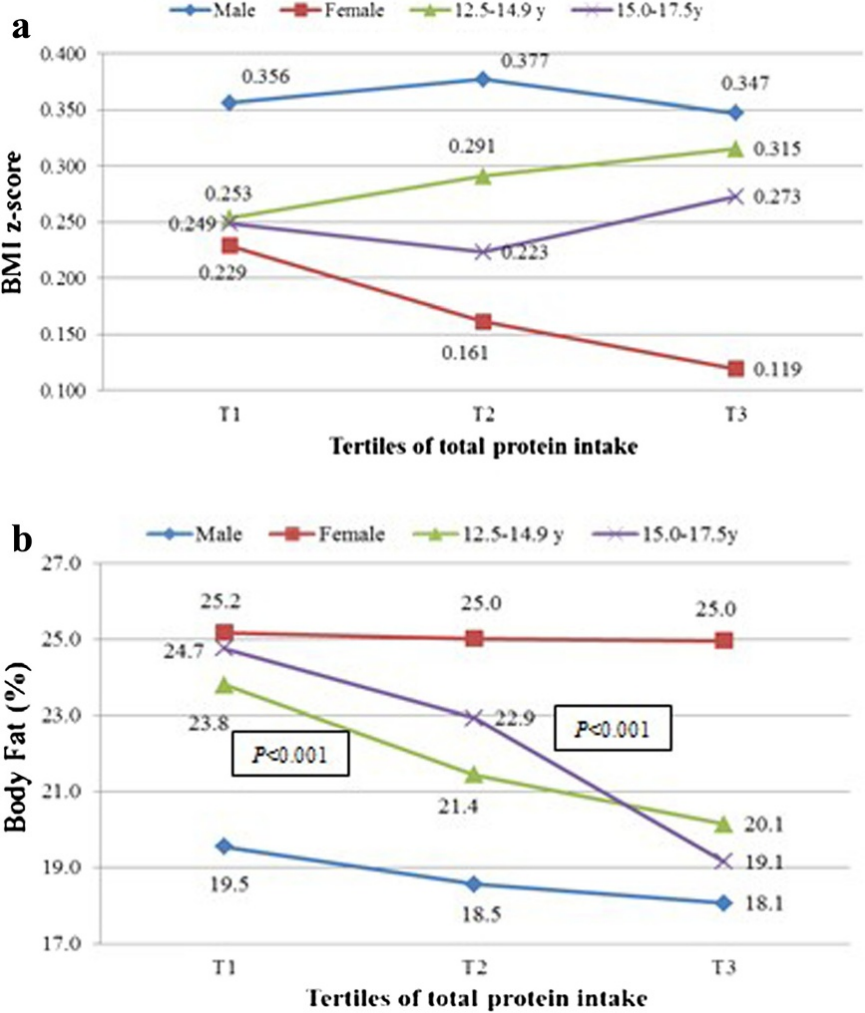
**Tertiles** ^**μ**^ **of total protein intake (g/d) and anthropometric indicators in adolescents participating in HELENA-CSS (n = 1804).** ^μ^Tertile 1 (T1): <81 g/d; tertile 2 (T2): 81 g/d to 103 g/d; tertile 3 (T3): ≥103 g/d.

Figure 1 shows a significant decline in BF% across the total protein tertiles (*P* < 0.001) by age. But no significance was observed in males and females. The results of the GLM multivariate analysis showed that crude BF% was inversely associated with absolute animal and plant protein in model 1, but crude BMI z-score and BF% were positively associated with animal protein (E%) (Table 4). Absolute animal protein intake was inversely associated with crude serum biomarkers including TC, TG, VLDL-C and leptin, but positively with serum fasting glucose. While absolute plant protein intake was inversely associated with crude TC, HDL-C, and leptin, but positively with serum fasting glucose. After adjustments for fat intake (Model 2), BMI z-score became positively associated with absolute animal protein intake, but several significant associations found in model 1 disappeared. Leptin kept to be inversely associated with absolute animal protein intake in model 2, and BF%, TC and HDL-C with absolute plant protein intake. Only serum HDL-C became positively associated with absolute animal protein intake, after further adjusting for confounding factors, PA and interaction factors (Model 3). Inverse associations were observed between BMI z-scores and BF%, and absolute plant protein intake. Whereas both BMI z-scores and BF% were positively associated with animal protein (E%). No biomarker was associated with percentage of energy intake derived from animal and plant protein (data not shown).

**Table 4:** **Associations between dietary animal and plant protein intakes (g/d and E%) and body composition of adolescents participating in the HELENA-CSS (n = 1804)**

Dependent variables ^μ^	Animal protein (g/d)				Plant protein (g/d)			
	β	SE	95% CI	***P***	β	SE	95% CI	***P***
BMI z-score								
Model 1	0.001	0.001	-0.001, 0.004	0.206	-0.002	0.002	-0.006, 0.002	0.414
Model 2	0.003	0.001	0.000, 0.006	0.023	0.000	0.002	-0.005, 0.004	0.847
Model 3	-0.000002	0.000001	-0.000007, 0.000	0.421	-0.012	0.005	-0.023, -0.001	0.027
Body fat (%)								
Model 1	-0.054	0.009	-0.071, -0.036	<0.001	-0.162	0.016	-0.194, -0.131	<0.001
Model 2	-0.009	0.010	-0.030, 0.011	0.386	-0.106	0.019	-0.144, -0.069	<0.001
Model 3	-0.000052	0.000018	-0.000087, - 0.000016	0.004	-0.139	0.040	-0.217, -0.060	0.001
	**Animal protein (E%)**				**Plant protein (E%)**			
BMI z-score								
Model 1	0.021	0.008	0.005, 0.038	0.011	0.012	0.019	-0.026, 0.050	0.533
Model 2	0.021	0.008	0.005, 0.037	0.011	0.008	0.020	-0.031, 0.046	0.692
Model 3	0.024	0.009	0.006, 0.043	0.010	-0.027	0.021	-0.067, 0.013	0.188
Body fat (%)								
Model 1	0.209	0.067	0.077, 0.341	0.002	0.124	0.156	-0.181, 0.429	0.426
Model 2	0.196	0.065	0.068, 0.325	0.003	-0.179	0.154	-0.482, 0.123	0.245
Model 3	0.168	0.070	0.030, 0.305	0.017	-0.229	0.151	-0.526, 0.068	0.130

SE, standard error of coefficient β; CI, confidence interval.

^μ^Model 1, unadjusted; model 2, adjusted for fat intake; model 3, model 2 further adjusted for age, sex, tanner stage, physical activity, country cluster, and interactions between potential confounding factors and animal / plant protein (separate model).

## Discussion

The HELENA study is the first large-scale European adolescent population-based dietary survey of 8 European countries providing data on the nutritional intake, status, main determinants of food choices and preferences among European adolescents. The current study is the first to provide information on intakes of total, animal and plant proteins and their associations with OB and cardio-metabolic indicators.

### Total energy and total, animal and plant protein intakes

The contribution of protein to energy intake in our study was similar to that reported in Greek and Italian adolescents, lower than that of Spanish peers (male: 17.2%, female: 17.8%) [42], but higher than adolescents in review studies of Western, Central and Eastern European countries [43–45]. In addition, total protein intake was reported to be slightly lower in Italian peers (male: 99 g/d, female: 82 g/d) [46], Spanish males (male: 105 g/d, female: 86 g/d) [42], and Western European adolescents [43, 45]. The adolescents in this study had much higher animal and plant protein intakes than those of Belgian peers (male: 52 g/d, female: 37 g/d; male: 30 g/d, female: 24 g/d, respectively) [43] and higher plant protein intake (male: 30 g/d, female: 25 g/d), but lower animal protein intake than Spanish peers (male: 74 g/d, female: 60 g/d).

### Associations between total, animal and plant protein intakes and cardio-metabolic indicators

Obese HELENA participants consumed more total protein than non-obese participants. Evidence from other European studies indicate higher contribution of animal sources [44, 47] to total protein and lower from plant protein consumptions [45], which might point to a relationship between increasing prevalence of OB in European adolescents. Our results suggest that increasing total protein intakes may be inversely associated with adolescents’ BF%, which can be explained by plant protein intakes being significantly inversely associated with BMI z-score and BF%, after adjustment for fat intake, PA and confounding factors. Consistent with our findings, observed benefits of increasing total and plant protein intakes on body composition [14, 48] could be attributed to the protein effect on increasing stimulated fat oxidation and building of lean body mass [49]. Conversely, the results of a previous randomized trial on obese adolescents (11–16 y) demonstrated that increasing protein consumption conferred no benefit on weight loss and body composition in the treatment of adolescent OB [12]. The different study design and target population might partly explain differences observed. Remarkably, the level of serum leptin was found to be extremely low among males in our study. High levels of leptin can easily be observed in female adolescents, because leptin was reported to play a critical role in the regulation of puberty, especially in females [50]. Serum leptin is proven to be related to BF% [51], and this might partly explain our finding on why females kept high BF% when increasing total protein intake, whereas BF% in males decreased gradually.

Evidence shows that plant protein from vegetables, fruits, and legumes not only improves body composition, but also results in lower body weight compared to animal protein [13, 52]. In our study, although animal protein intake was found to be weakly inversely associated with BF%, animal protein (E%) was observed to be positively associated with BF%. Previous studies concluded that total and animal protein intakes might be responsible for increasing body weight and BMI in adolescents [12, 13]. Mirkopoulou *et al.* suggested that extremely high protein intakes, animal protein in particular, might increase the risk of adolescents’ OB due to higher energy consumption [53]. Furthermore, the results of a longitudinal study suggested that a high animal protein intake in mid-childhood might be associated with an earlier pubertal growth and spurt peak height velocity, whereas a higher plant protein intake could delay puberty [54]. On the contrary, some studies disagreed the above hypothesis of increased intake of total and animal protein resulting in decreasing the risk of OW and OB [55, 56] by affecting the appetite. A randomized 8-weeks parallel intervention trial suggested that seafood protein sources from cod and salmon were efficient to treat OB because of caloric restriction and lower saturated fatty acids intake [55]. Therefore, the amount of total, animal and plant proteins in the diet may be a critical factor on prevention against OW and OB.

Evidence also shows that increasing protein intake results in improvement of serum lipids [57]. Plant protein based diets in childhood could be responsible for lowering the risk of MetS and its consequence in the adulthood [58]. In the current study, only serum HDL-C was found to be weakly positively associated with animal protein intake. The increases in HDL-C might possibly be explained by the inverse association of animal protein intake with BF%. Mirkopoulou *et al.* reported that no association with blood lipid profile was observed in Greek adolescents [53], supporting most of our results, as similarities in the study design and target population might explain similarities in observations. Some cross-sectional studies showed that plant based diets were associated with more favourable lipid levels in adolescents by lowering TC and LDL-C, but increasing the HDL-C levels [17, 59], whereas high intakes derived from animal sources were associated with an increased risk of MetS [15]. However, it has to be considered that adolescence is a critical period with inevitable increases in energy and nutrient intakes to regulate hormone balances resulting in physical, behaviour and social development. Leptin is a protein hormone that has a key role in regulating energy intake and energy expenditure, including appetite in the longer term [60, 61]. In the current study, no significance of serum leptin was found in model 3, but it was negatively associated with animal and plant protein intakes in model 1 and model 2, respectively. The status of statistical significance between serum leptin and plant protein intake changed in the model 2 compared to model 1 due to fat intake. In addition, fat intake can be a critical factor for the serum lipid profile and plant protein intake. No study has provided evidence on clear mechanisms, though it is possible that plant protein intake might stimulate serum leptin via homeostasis impacting on body weight and BF%. In addition, female, OW and obese adolescents in particular, during puberty might most likely underestimate energy and dietary intakes, which may bias the associations. Confounding factors, such as gender, age, Tanner stage and region, may account for some unexpected findings, serum biomarkers in particular.

### Strengths and limitations

This European nutrition survey is the first large-scale study among European adolescents that used a standardized approach accross 8 participating centers. Additionally, it is the first study evaluating total, animal and plant protein intakes in European adolescents stratified by gender and age, and investigating associations with anthropometry and serum biomarkers as studies with the same standardised methodology across European countries are limited.

The current study has also some limitations including the dietary assessment method used to assess diet that only included dietary information of two non-consecutive days. The 24-h dietary recall method does not allow quantifying proportions of non-consumers for particular food items, especially for those less frequently consumed. In order to decrease the influence of such limitation, nutrient intakes were corrected for within- person variability by applying the MSM method. Moreover, accuracy of collected data relies on the individual’s ability to remember foods and beverages consumed in the past 24 hours, and might, therefore, be biased towards misreporting. In this respect, the 24-h dietary recalls were performed through computer-assisted HELENA-DIAT software to standardize the recall procedures as much as possible. Food pictures, showing daily foods consumed by European adolescents, were used in order to facilitate the participants to recall the potion size of the foods consumed in the previous days, which assisted participants and interviewers in accurately assessing the consumed amounts. The same food composition table for conversion of food intake data to estimated nutrient intakes was used for all survey centres. In this way, differences in definitions, analytical methods, units and modes of expression were overcome. However, missing foods of protein contents in the BLS table were calculated via recipes or taken from local food composition tables. In addition, the small sample size of serum biomarkers may also be a potential influencing factor leading to weak linear relationship between animal and plant protein intakes and serum biomarkers. Furthermore, the cross-sectional study design of this study cannot assess causality between health outcomes and dietary intakes.

### Recommendations

Protein is critical for the development of bone and muscle mass, and health in adolescents. An increased protein intake is one of the most common approaches to the dietary management of OB and related chronic diseases. However, extra high protein intake can result in side-effects due to imbalance in energy intake and food consumption. The findings of current study indicate that plant protein had more protective effect against OB compared to animal protein, although HDL-C was found to be weakly positively associated with absolute animal protein intake. We noticed that participants exceeded protein intake based on WHO requirement, and almost 2/3 sources were from animal origin rather than from plants, which may influence body weight and body composition. The findings of our study highlight that future public health policies and school policies need to be developed and implemented to help establishing healthy food preferences, and adjusting food concepts and dietary behaviors in adolescents. Possible prevention strategies could include the development of multicomponent school-based interventions combining education and environmental changes towards increased intakes of plant proteins from legumes and vegetables.

## Conclusion

The total protein intake of European adolescents exceeded the recommendations and animal proteins contribute most to the energy intake derived from total protein intake. Total and animal protein intake and E% derived from protein intake were higher in obese subjects. A negative association of total protein intake was found with BF%. GLM multivariate analysis indicates inverse associations, on one hand, between BMI z-score and plant protein intake, and on the other hand between BF% and animal and plant protein intakes. Both BMI z-score and BF% were positively associated with animal protein (E%). In conclusion our findings suggest that plant protein intakes may play a role in preventing OB among European adolescents. Further longitudinal studies should be conducted to investigate these potential beneficial effects of plant protein intakes in the prevention of OB and related chronic diseases.

## Abbreviations

BF%: Body fat percentage
BMI: Body mass index
BMI z-score: Standard deviation score of BMI
BLS: Bundeslebensmittelschlüssel, the German food code and nutrient database
CRP: C-reactive protein
EAR: Estimated average requirement
EFSA: European food safety authority
HDL-C: High-density lipoprotein cholesterol
HELENA-CSS: The healthy lifestyle in Europe by nutrition in adolescence-cross sectional study
IOTF: The international obesity task force
LDL-C: Low-density lipoprotein cholesterol
MetS: Metabolic syndrome
MSM: The multiple source method
NEVO: Dutch food composition table
NUBEL: Belgium food composition table
NW: Normal weight
OB: Obesity
OW: Overweight
PA: Physical activity
PRI: Population reference intake
SES: Socioeconomic status
TC: Total cholesterol
TG: Triglyceride
VLDL-C: Very low-density lipoprotein cholesterol
USDA: The USDA national nutrient database
UW: Underweight
WHO: World health organization
YANA-C: The young adolescents’ nutrition assessment on computer.
